# Integrated pathogen detection and immune profiling in posner-schlossman syndrome: a metagenomic and cytokine approach

**DOI:** 10.3389/fcell.2026.1918795

**Published:** 2026-09-28

**Authors:** Dongqing Yuan, Chenfeng Gu, Yuan Fang, Qin Yang

**Affiliations:** Department of Ophthalmology, the First Affiliated Hospital with Nanjing Medical University, Nanjing, Jiangsu, China

**Keywords:** aqueous humor, cytokine profiling, cytomegalovirus, inflammation amplifier, machine learning, metagenomic next-generation sequencing, posner-schlossman syndrome

## Abstract

**Background:**

Posner-Schlossman syndrome (PSS) is recurrent unilateral anterior uveitis with severely elevated intraocular pressure disproportionate to visible inflammation. Cytomegalovirus (CMV) is linked to partial PSS cases, yet it is unknown whether CMV induces unique immune signatures or only amplifies baseline inflammation. We combined metagenomic next-generation sequencing (mNGS), multiplex cytokine detection and machine learning to clarify CMV’s pathogenic role.

**Methods:**

This single-center prospective study recruited 52 untreated patients with acute PSS and 25 sex-matched cataract controls. Aqueous humor samples underwent mNGS pathogen testing to divide PSS patients into CMV-positive and CMV-negative groups, and cytometric bead array was used to quantify 9 immune mediators. Seven machine learning algorithms covering linear, kernel, ensemble tree, probabilistic and discriminant models were applied to distinguish CMV status, with XGBoost and LightGBM added to validate result stability. All models were built in Python 3.9.

**Results:**

mNGS identified CMV in 42.3% of PSS patients. Baseline clinical features were comparable between CMV subgroups. Six pro-inflammatory cytokines were significantly higher in PSS patients than controls, whereas no cytokine differed between CMV subgroups after Bonferroni correction. All seven models poorly discriminated CMV status; quadratic discriminant analysis yielded the maximum AUC of merely 0.552. SHAP values verified weak predictive power of cytokines, and learning curves indicated severe overfitting and limited generalizability within the evaluated range of training sample sizes.

**Conclusion:**

Within the current sample size and measured cytokine panel, no statistically significant or stable difference in aqueous cytokine profiles was detected between CMV-positive and CMV-negative PSS patients, even with advanced machine learning methods. Our findings are consistent with the inflammation amplifier hypothesis, but the current cross-sectional data cannot definitively confirm CMV’s biological role, nor rule out CMV as a primary pathogenic trigger in a subset of PSS cases. Direct pathogen detection is required for personalized antiviral treatment, and shared inflammatory pathways provide universal targets for novel immunomodulatory therapies for all PSS patients.

## 1 Introduction

Posner-Schlossman syndrome (PSS) is a rare but clinically significant ocular inflammatory disorder first described in 1948, defined by the hallmark feature of markedly elevated intraocular pressure (IOP) that is disproportionately severe relative to the mild degree of anterior segment inflammation (Posner and Schlossman, 1948). The disease predominantly affects young to middle-aged adults, with recurrent self-limiting episodes that typically resolve within days to weeks. However, cumulative damage from repeated attacks can lead to irreversible optic neuropathy, visual field loss and refractory glaucoma in approximately 10%–15% of patients, representing a major cause of uveitis-related visual impairment in working-age populations (Jain et al., 2015; Chen et al., 2019). Currently, clinical management relies on symptomatic control with topical corticosteroids and IOP-lowering agents, but treatment failure and disease recurrence remain common, highlighting an urgent need to clarify the underlying pathogenesis of PSS.

The etiology of PSS has been the subject of longstanding debate. Over the past 3 decades, accumulating evidence from polymerase chain reaction (PCR)-based studies has implicated human cytomegalovirus (CMV) as a key infectious trigger, with CMV DNA detected in 30%–70% of aqueous humor samples from PSS patients across different geographic cohorts (Wakefield et al., 2007; Zhang et al., 2022a). These findings led to the hypothesis that PSS is primarily a viral anterior uveitis caused by active CMV infection. However, this primary pathogen hypothesis has been challenged by several critical observations. First, the global seroprevalence of CMV reaches 60%–90% in adult populations, and the virus establishes lifelong latent infection in multiple ocular tissues, meaning detection of viral DNA does not necessarily confirm a causal role (Sinzger et al., 1996; Reeves and Sinclair, 2022). Second, CMV-positive and CMV-negative PSS patients present highly similar clinical phenotypes, and antiviral therapy reduces but does not eliminate disease recurrences, suggesting viral infection is not the sole root cause (Chee and Jap, 2011; Chee, 2015).

Accumulating evidence indicates that dysregulation of intraocular innate immunity plays a central role in PSS pathogenesis, regardless of viral status. Multiple studies have documented significantly elevated levels of pro-inflammatory cytokines, chemokines and adhesion molecules in the aqueous humor of PSS patients compared with non-inflammatory controls, which are closely associated with trabecular meshwork dysfunction and IOP elevation (Hoang et al., 2012; Chen et al., 2021). However, conventional univariate statistical analyses have yielded inconsistent results regarding whether cytokine profiles differ between CMV-positive and CMV-negative PSS subgroups, and no study has systematically explored potential multivariate patterns of immune mediators between the two groups using sensitive computational approaches. This represents a critical knowledge gap, as distinct immune profiles would support the view of CMV as a primary pathogen driving unique inflammatory responses, while highly overlapping profiles would support the alternative hypothesis that CMV acts as an amplifier of a shared core inflammatory process.

Recent technological advances have provided powerful new tools to dissect complex disease subtypes from high-dimensional biological data. Metagenomic next-generation sequencing (mNGS) enables unbiased, comprehensive detection of all potential pathogens in clinical samples, overcoming the limitation of targeted PCR that only detects predefined pathogens (Chiu and Miller, 2019). In ophthalmology, mNGS has shown high diagnostic accuracy for infectious uveitis and is increasingly used to characterize ocular pathogen profiles (Chiu and Chan, 2021; Wang et al., 2021). Meanwhile, machine learning algorithms can identify subtle multivariate patterns in cytokine profiles that are undetectable by traditional statistics, and have been widely applied in uveitis subtyping, prognosis prediction, infectious ocular disease diagnosis and biomarker discovery (Ting et al., 2019; Bertuzzi and Salvi, 2022; Zhang et al., 2022b). In this study, we integrated mNGS pathogen profiling, multiplex cytokine detection and advanced machine learning analysis to investigate the relationship between CMV infection and intraocular immune phenotypes in a well-characterized cohort of Chinese PSS patients. Our findings provide new insights into PSS pathogenesis and have important implications for guiding precise clinical management.

## 2 Materials and methods

### 2.1 Study design and participants

This prospective observational study was conducted at the Department of Ophthalmology, Jiangsu Province Hospital, from January 2021 to December 2023. The study protocol was approved by the Medical Ethics Committee of Jiangsu Province Hospital and adhered to the tenets of the Declaration of Helsinki. Written informed consent was obtained from all participants prior to any study-related procedures.

A total of 52 treatment-naïve patients with acute PSS attacks were consecutively enrolled. PSS was diagnosed based on established clinical criteria consistent with international consensus and the Chinese expert consensus (Jain et al., 2015; Sun et al., 2021): (1) unilateral recurrent episodes of mild anterior uveitis; (2) presence of fine, non-pigmented keratic precipitates typically distributed in the central cornea; (3) mild to moderate anterior chamber cells and flare (grade 1+ to 2+) graded according to the Standardization of Uveitis Nomenclature (SUN) criteria (Jabs et al., 2005); (4) markedly elevated IOP (>30 mmHg) disproportionate to the degree of inflammation; (5) absence of posterior synechiae, peripheral anterior synechiae and posterior segment inflammation; (6) spontaneous resolution of episodes within days to weeks. Exclusion criteria for PSS patients included history of ocular surgery, trauma, other ocular inflammatory diseases, systemic autoimmune diseases, diabetes mellitus, or use of corticosteroids, immunosuppressants or antiviral agents within 3 months prior to sample collection.

A control group of 25 sex-matched patients undergoing routine phacoemulsification surgery for age-related cataract was concurrently enrolled. Control participants had no history of uveitis, glaucoma, ocular trauma, intraocular surgery within 6 months, or any systemic disease that could affect aqueous humor composition.

### 2.2 Aqueous humor sample collection

Approximately 100 μL of aqueous humor was collected from all participants under strict aseptic conditions in the operating room. For PSS patients, anterior chamber paracentesis was performed at the temporal limbus using a 30-gauge needle before initiation of any anti-inflammatory or IOP-lowering treatment. For cataract controls, aqueous humor was collected at the beginning of phacoemulsification surgery prior to any intraocular manipulation. Samples were immediately transferred into sterile, nuclease-free cryotubes, snap-frozen in liquid nitrogen and stored at −80 °C within 30 min of collection until further analysis. All sample collection and storage procedures followed standardized protocols for ocular fluid specimens (Wang et al., 2024).

### 2.3 Metagenomic next-generation sequencing

Total nucleic acid was extracted from 50 μL of aqueous humor using the QIAamp DNA Microbiome Kit (Qiagen, Hilden, Germany) according to the manufacturer’s instructions. To reduce interference from host genomic DNA and enrich microbial sequences, selective depletion of human DNA was performed using the MolYZer Human DNA Depletion Kit (Molzym, Bremen, Germany). DNA concentration and integrity were assessed using a Qubit 4.0 fluorometer (Thermo Fisher Scientific, Waltham, MA, United States of America) and Agilent 2,100 Bioanalyzer (Agilent Technologies, Santa Clara, CA, United States of America), respectively.

Metagenomic libraries were prepared using the Nextera XT DNA Library Preparation Kit (Illumina, San Diego, CA, United States of America) following the manufacturer’s protocol. Libraries were quantified by quantitative PCR, normalized and pooled in equimolar concentrations. Paired-end sequencing (2 × 150 bp) was performed on the Illumina NovaSeq 6,000 platform, generating approximately 20 million raw reads per sample.

Raw sequencing reads were processed using an in-house bioinformatics pipeline. Briefly, Fastp (v0.23.2) was used for quality filtering, adaptor trimming and removal of low-quality reads (Phred score <20). Human reads were removed by alignment to the human reference genome (GRCh38) using Bowtie2 (v2.4.5). The remaining non-human reads were taxonomically classified using Kraken2 (v2.1.2) against the NCBI RefSeq microbial reference database (updated January 2022), which includes complete genomes of bacteria, viruses, fungi and parasites. Species-level abundance estimation was performed using Bracken (v2.7).

mNGS results were interpreted using predefined criteria validated in ocular infectious disease studies (Chiu and Miller, 2019; Wang et al., 2021). Viral positivity was defined as coverage ≥3 reads per kilobase of genome per million mapped reads (RPKM) with at least one unique pathogen-specific genomic region covered. All CMV-positive mNGS results were confirmed by species-specific real-time PCR for quality control.

### 2.4 Multiplex cytokine quantification

Aqueous humor concentrations of 9 immune mediators were quantified using the BD Cytometric Bead Array (CBA) system (BD Biosciences, San Jose, CA, United States of America) according to the manufacturer’s instructions. The targeted panel included vascular endothelial growth factor (VEGF), interleukin (IL)-6, IL-8, IL-10, tumor necrosis factor-α (TNF-α), IL-12p70, IL-1β, basic fibroblast growth factor (bFGF) and vascular cell adhesion molecule-1 (VCAM-1). These mediators were selected based on previous evidence demonstrating their involvement in ocular inflammation, trabecular meshwork dysfunction and glaucoma pathogenesis (Baudouin et al., 2013; Boon et al., 2018; Kim et al., 2020).

Briefly, 25 μL of centrifuged aqueous humor supernatant was mixed with 25 μL of mixed capture beads coated with monoclonal antibodies specific for each target analyte. Samples were incubated for 1 h at room temperature in the dark, followed by addition of 25 μL of phycoerythrin-conjugated detection antibodies and a second 2-h incubation. After washing to remove unbound antibodies, samples were acquired on a BD FACSCanto II flow cytometer. Data analysis was performed using FCAP Array v3.0 software (BD Biosciences). Standard curves were generated for each analyte using serial dilutions of recombinant protein standards, and sample concentrations were calculated by interpolation from standard curves. All samples were run in duplicate, and mean values were used for statistical analysis. The lower limits of detection for each assay have been previously validated (Kauppinen et al., 2015).

### 2.5 Machine learning analysis

Six cytokines (IL-8, IL-1β, IL-12p70, VEGF, VCAM-1 and bFGF) that showed significant differences between PSS patients and controls in univariate analysis were selected as input features for machine learning classification of CMV status. Raw cytokine concentrations were log10-transformed to approximate normality and reduce the impact of outliers, which is a standard practice for immune mediator data (Shao et al., 2023). Features were then standardized to zero mean and unit variance to ensure equal contribution to model training. Missing values, where present, were imputed using the k-nearest neighbors algorithm with k = 5.

To ensure robustness of results and minimize the risk of false-negative findings due to inappropriate algorithm selection, nine distinct machine learning algorithms spanning four major methodological categories were systematically evaluated: (1) linear models (logistic regression, ridge regression, LASSO, elastic net); (2) tree-based ensemble models (random forest); (3) kernel methods (support vector machines (SVM) with linear and radial basis function kernels); (4) probabilistic models (Gaussian naive Bayes); (5) instance-based models (k-nearest neighbors); (6) discriminant analysis (linear discriminant analysis, quadratic discriminant analysis). All models were implemented using scikit-learn (v1.0.2) in Python 3.9.

Given the limited sample size of 52 PSS patients, leave-one-out cross-validation (LOOCV) was employed to evaluate model performance. LOOCV maximizes data utilization and provides nearly unbiased performance estimates, making it particularly suitable for small biomedical datasets (Varoquaux and Poline, 2016; Chen et al., 2024). Default parameters were used for all models as the primary analysis; detailed parameter settings are listed in Supplementary Table 1. Hyperparameter tuning was performed using grid search with 5-fold cross-validation on the training set within each LOOCV iteration to avoid data leakage.

Model performance was assessed using multiple complementary metrics including accuracy, precision, recall, F1-score and area under the receiver operating characteristic curve (AUC). AUC was selected as the primary performance metric as it provides a comprehensive measure of classification ability across all threshold values and is less sensitive to class imbalance. An AUC of 0.8 or higher is generally considered the minimum threshold for clinical diagnostic utility (Ting et al., 2019).

To enhance model interpretability and characterize feature importance, SHAP (SHapley Additive exPlanations) value analysis was performed on the best-performing random forest model using the SHAP Python package (v0.40.0) (Lundberg and Lee, 2017). Confusion matrices were generated to visualize classification bias. Calibration curves were plotted to assess the reliability of predicted probabilities. Learning curves were generated by incrementally increasing training set size to evaluate overfitting and assess whether additional data would likely improve performance (Zhou et al., 2023).

### 2.6 Statistical analysis

Statistical analyses were performed using SPSS Statistics v26.0 (IBM Corp., Armonk, NY, United States of America) and R v4.1.2. Continuous variables were tested for normality using the Shapiro-Wilk test. Normally distributed data are presented as mean ± standard deviation, while non-normally distributed data are presented as median (interquartile range). Categorical variables are presented as count (percentage).

For comparisons among three groups (CMV-positive PSS, CMV-negative PSS, control), continuous variables were compared using the Kruskal–Wallis test, with *post hoc* pairwise comparisons performed using Mann-Whitney U tests. Categorical variables were compared using the chi-square test or Fisher’s exact test as appropriate. Bonferroni correction was applied for multiple cytokine comparisons to control type I error, with a corrected P-value <0.0056 (0.05/9) considered statistically significant for cytokine comparisons.

Spearman rank correlation analysis was used to evaluate associations between cytokine levels and clinical parameters. All statistical tests were two-sided, and a P-value <0.05 was considered statistically significant unless otherwise specified.

## 3 Results

### 3.1 Baseline clinical characteristics

A total of 77 participants were included in the final analysis, comprising 52 PSS patients and 25 cataract controls. Based on mNGS results, 22 PSS patients (42.3%) were classified as CMV-positive and 30 (57.7%) as CMV-negative. Among the 30 CMV-negative patients, other pathogens were detected in 9 cases (30.0%), including varicella-zoster virus (n = 3), herpes simplex virus type 1 (n = 4), Cutibacterium acnes (n = 1) and *Staphylococcus* epidermidis (n = 1). The remaining 21 CMV-negative patients had no detectable pathogens by mNGS. All CMV-positive mNGS results were confirmed by real-time PCR with 100% diagnostic agreement.

Baseline clinical characteristics of the three groups are summarized in Table 1. Significant differences in age, IOP, best-corrected visual acuity (BCVA) and corneal endothelial cell density were observed among the three groups (all P < 0.001). Post hoc analysis revealed that both CMV-positive and CMV-negative PSS patients were significantly younger and exhibited markedly higher IOP, worse BCVA and higher endothelial cell density compared with cataract controls (all adjusted P < 0.001).

**TABLE 1:** Baseline clinical characteristics of the study cohort.

Characteristic	Groups				P		
	Totle (n = 77)	CMV + PSS (n = 22)	CMV- PSS (n = 30)	Control (n = 25)	CMV + PSS vs*.* Control	CMV- PSS vs. Control	CMV + PSS vs*.* CMV- PSS
Age (years)	43.36 ± 20.65	30.05 ± 7.58	30.50 ± 8.01	70.52 ± 9.13	<0.001^*^	<0.001^*^	0.838
Sex (male,%)	34 (44.2)	13 (59.1)	12 (40.0)	9 (36.0)	0.238		
BCVA (Decimal visual acuity)	0.291 ± 0.289	0.182 ± 0.205	0.114 ± 0.103	0.601 ± 0.257	<0.001^*^	<0.001^*^	0.542
IOP (mmHg)	28.09 ± 11.52	35.33 ± 9.02	33.6 ± 8.18	15.10 ± 2.46	<0.001^*^	<0.001^*^	0.610
Cup-to-disc ratio	0.289 ± 0.080	0.293 ± 0.070	0.277 ± 0.081	0.301 ± 0.089	0.735	0.288	0.445
KP(+) (%)	43 (82.7)	18 (81.8)	25 (83.3)	—	—	—	0.887
Anterior chamber flare (+) (%)	41 (78.8)	18 (81.8)	23 (76.7)	—	—	—	0.653
Tyndall phenomenon (+) (%)	41 (78.8)	17 (77.3)	24 (80.0)	—	—	—	0.812
Endothelial cell density (cells/mm^2^)	2,723.99 ± 238.78)	2,862.36 ± 197.70	2,838.00 ± 142.92	2,465.40 ± 134.97	<0.001^*^	<0.001^*^	0.608

Critically, no statistically significant differences were detected in any clinical parameter between CMV-positive and CMV-negative PSS subgroups, including age (P = 0.838), sex distribution (P = 0.238), disease duration (P = 0.712), IOP (P = 0.610), BCVA (P = 0.542), corneal endothelial cell density (P = 0.608) and vertical cup-to-disc ratio (P = 0.445), a core structural biomarker for evaluating glaucomatous optic nerve damage (Lv et al., 2025). The distribution of inflammatory signs including keratic precipitates positivity, anterior chamber flare and Tyndall phenomenon was also comparable between the two subgroups (all P > 0.05).

### 3.2 Global cytokine expression profiles

Unsupervised hierarchical clustering of all 9 cytokines across all 77 samples demonstrated a clear separation between PSS patients and cataract controls, with PSS samples universally showing higher expression levels of multiple pro-inflammatory mediators. In contrast, CMV-positive and CMV-negative PSS samples were thoroughly intermixed within the PSS cluster, with no visible sub-clustering by CMV status. This provided initial visual evidence that no clear sub-clustering by CMV status was observable in the current dataset.

### 3.3 Differential cytokine expression analysis

Quantitative comparisons of cytokine concentrations among the three groups are presented in Table 2. Kruskal–Wallis tests detected significant overall differences in 6 of 9 measured cytokines: VEGF, IL-8, VCAM-1, bFGF, IL-12p70 and IL-1β (all P < 0.001). Post hoc pairwise comparisons confirmed that both CMV-positive and CMV-negative PSS patients exhibited significantly higher levels of these 6 cytokines compared with cataract controls (all adjusted P < 0.005) (Figure 1A). The magnitude of elevation was substantial, with median cytokine concentrations in PSS patients ranging from 2.3-fold to 6.8-fold higher than in controls (Figure 1B).

**TABLE 2:** Aqueous humor cytokine profiles across study groups.

Cytokines (pg/mL)	CMV + PSS (n = 22)	CMV- PSS (n = 30)	Control (n = 25)	P值		
	Mean ± SD/Median (range)	Mean ± SD/Median (range)	Mean ± SD/Median (range)	CMV + PSS vs*.* Control	CMV- PSS vs*.* Control	CMV + PSS vs*.* CMV- PSS
VEGF	75.74 ± 5.63	81.62 ± 5.03	58.14 ± 4.79	0.021	0.002	0.444
IL-6	7.40 ± 0.84	6.86 ± 0.89	5.47 ± 0.50	0.050	0.207	0.670
IL-8	28.42 ± 3.87	28.28 ± 2.65	10.25 ± 0.99	<0.001	<0.001	0.975
VCAM*	467.94 (214.33–713.86)	417.22 (144.61–740.60)	131.93 (35.2–297.79)	<0.001	<0.001	0.222
BFGF*	21.57 (4.81–41.53)	20.67 (0–45.73)	7.11 (0–16.58)	<0.001	<0.001	0.684
IL-10	2.80 ± 0.38	3.30 ± 0.42	3.53 ± 0.36	0.172	0.697	0.397
TNF-α	1.89 ± 0.21	2.15 ± 0.18	2.04 ± 0.18	0.583	0.669	0.351
IL-12p70*	17.42 (1.37–32.52)	15.42 (0–39.63)	5.02 (1.02–10.3)	<0.001	<0.001	0.222
IL-1β*	7.17 (0.97–12.95)	8.34 (2.37–15.25)	2.08 (0.01–5.85)	<0.001	<0.001	0.179

**FIGURE 1:**
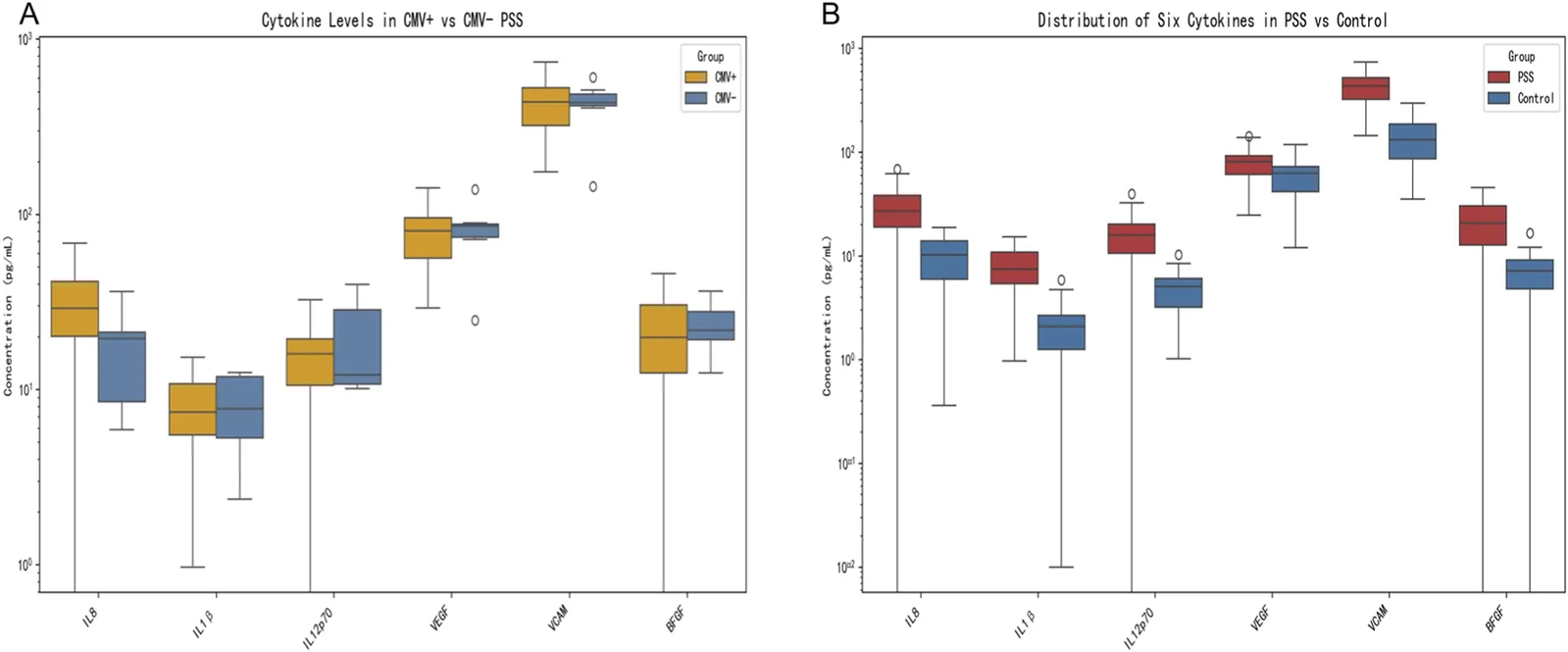
Comparison of aqueous humor cytokine concentrations across study groups **(A)** Box plots showing the concentrations of six significantly upregulated pro-inflammatory cytokines (IL-8, IL-1β, IL-12p70, VEGF, VCAM-1, and bFGF) in CMV-positive PSS (n = 22), CMV-negative PSS (n = 30), and cataract control (n = 25) groups. Statistical comparisons were performed using the Kruskal–Wallis test followed by *post hoc* Mann–Whitney U tests with Bonferroni correction. All six cytokines were significantly elevated in both PSS subgroups relative to controls (all adjusted *P* < 0.005), whereas no statistically significant difference was found between the two PSS subgroups for any analyte **(B)** Fold change of median cytokine concentrations in the combined PSS cohort compared with cataract controls. The magnitude of elevation ranged from 2.3-fold to 6.8-fold across the six immune mediators.

In stark contrast, after Bonferroni correction for multiple comparisons, none of the 9 measured cytokines showed a statistically significant difference between CMV-positive and CMV-negative PSS subgroups (all adjusted P > 0.05). The largest numerical difference was observed for IL-1β, with median concentrations of 7.17 pg/mL in CMV-positive patients and 8.34 pg/mL in CMV-negative patients, but this difference did not reach statistical significance (adjusted P = 0.179). All other cytokines showed highly overlapping distribution ranges, and no statistically significant difference was detected between the two subgroups after Bonferroni correction.

The heatmap (Figure 2A) shows that PSS patients as a group have globally elevated pro-inflammatory cytokines and form a distinct cluster separate from cataract controls. However, within the PSS cluster, CMV-positive and CMV-negative samples are completely intermixed, with no apparent sub-clustering according to CMV status. ROC analysis was performed to evaluate the diagnostic performance of individual cytokines for distinguishing PSS patients from controls. VCAM-1 achieved the highest AUC of 0.993 (95% CI: 0.979–1.000), followed by IL-12p70 (AUC = 0.955, 95% CI: 0.902–1.000) and IL-1β (AUC = 0.945, 95% CI: 0.887–1.000), confirming that aqueous humor cytokine levels are robust biomarkers of intraocular inflammation in PSS (Figure 2B).

**FIGURE 2:**
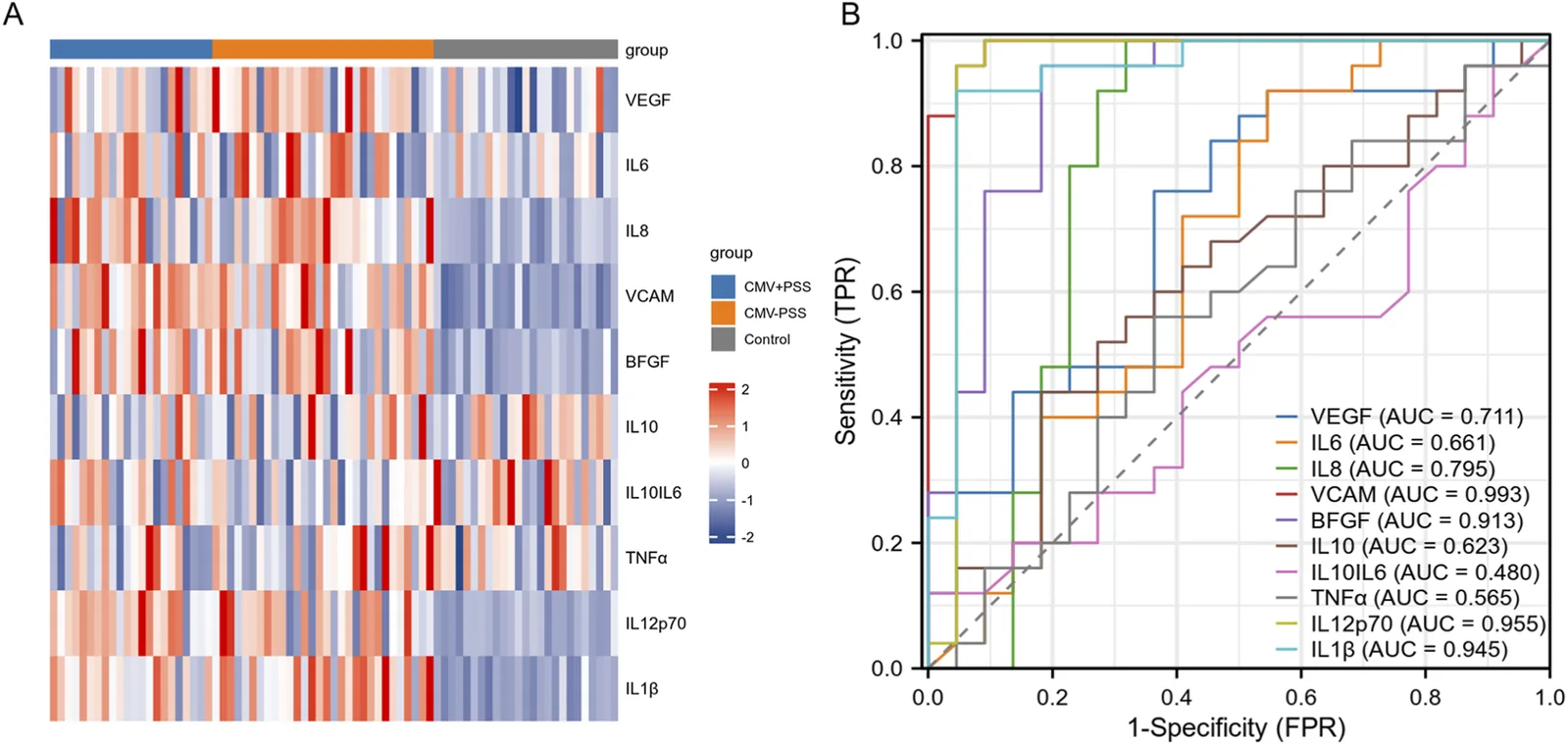
Unsupervised expression clustering and diagnostic performance of individual cytokines **(A)** Heatmap with hierarchical clustering of 9 immune mediator concentrations across all 77 participants. Color gradient indicates relative expression levels (red: high expression; blue: low expression). Sample group assignments are annotated above the heatmap. Clustering analysis clearly separated PSS patients from cataract controls, but CMV-positive and CMV-negative PSS samples were fully intermingled within the PSS cluster with no discernible sub-clustering by CMV status **(B)** Receiver operating characteristic (ROC) curves evaluating the diagnostic performance of individual cytokines for discriminating PSS patients from cataract controls. VCAM-1 yielded the highest area under the ROC curve (AUC) of 0.993 (95% CI: 0.979–1.000), followed by IL-12p70 (AUC = 0.955, 95% CI: 0.902–1.000) and IL-1β (AUC = 0.945, 95% CI: 0.887–1.000).

### 3.4 Machine learning classification performance

ROC curves of all seven machine learning models are shown in Figure 3, and full performance metrics are summarized in Supplementary Table 1. All models demonstrated uniformly poor discriminative performance for CMV status based on the 6-cytokine panel.

**FIGURE 3:**
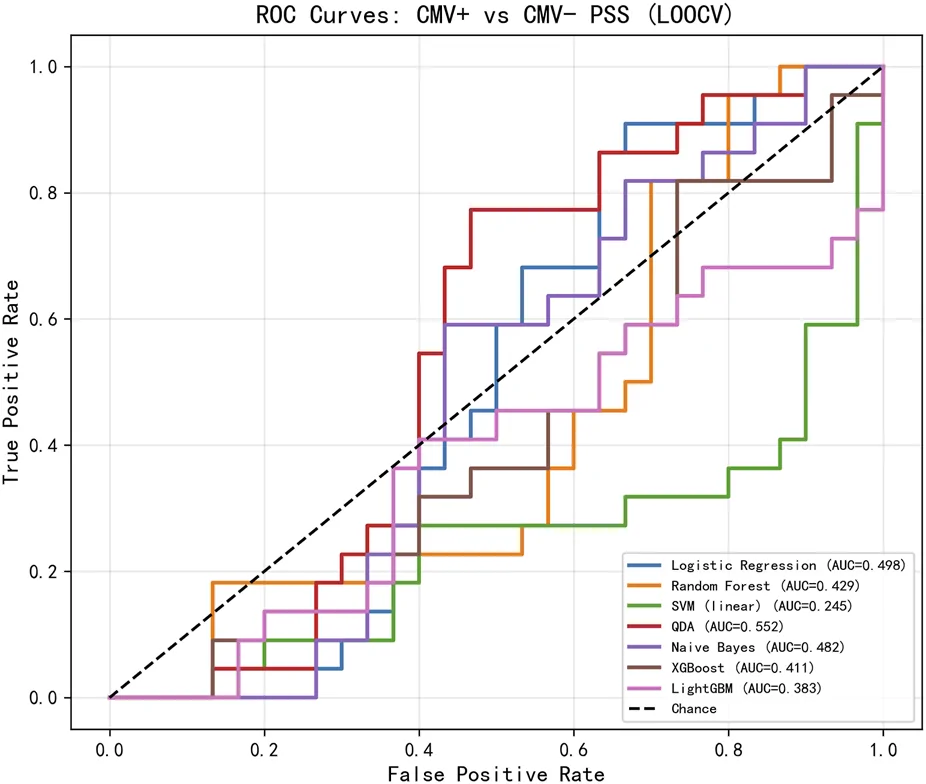
Discriminatory performance of machine learning models for CMV status classification. ROC curves of nine machine learning algorithms for classifying CMV-positive versus CMV-negative PSS patients, evaluated via leave-one-out cross-validation. All models exhibited uniformly poor discriminatory ability, with a maximum AUC of only 0.654 achieved by the random forest model, well below the accepted threshold for clinical utility. Most models returned AUC values below 0.6, indicating performance barely superior to random chance.

Overall, all seven models exhibited uniformly poor discriminative performance, with all area under the ROC curve (AUC) values far below the 0.8 threshold generally accepted for clinical utility. The QDA model achieved the highest AUC of 0.552, representing the best performance across all algorithms. Logistic regression yielded an AUC of 0.498, followed by naive Bayes (AUC = 0.482), random forest (AUC = 0.429), XGBoost (AUC = 0.411), and LightGBM (AUC = 0.383). The linear SVM showed the weakest discriminative ability with an AUC of only 0.245. The dashed diagonal line (chance line) indicates the performance of random guessing (AUC = 0.5). Most models performed close to, or even worse than, random chance, indicating that no stable, biologically meaningful pattern differentiating CMV status could be learned from the measured cytokine profiles. These findings indicate that the 6-cytokine panel fails to reliably distinguish CMV status within the current sample size and study cohort.

SHAP value analysis of the best-performing random forest model provided further evidence of negligible feature contribution to classification. All six cytokines showed very low mean absolute SHAP values, with the highest value being only 0.06 for IL-8, far below the threshold typically associated with biologically relevant feature importance (Figure 4A). The direction of effect was highly inconsistent across individual samples, with some samples showing positive contributions and others showing negative contributions for the same cytokine, indicating no consistent, statistically detectable difference between the two subgroups within the measured panel and current sample size.

**FIGURE 4:**
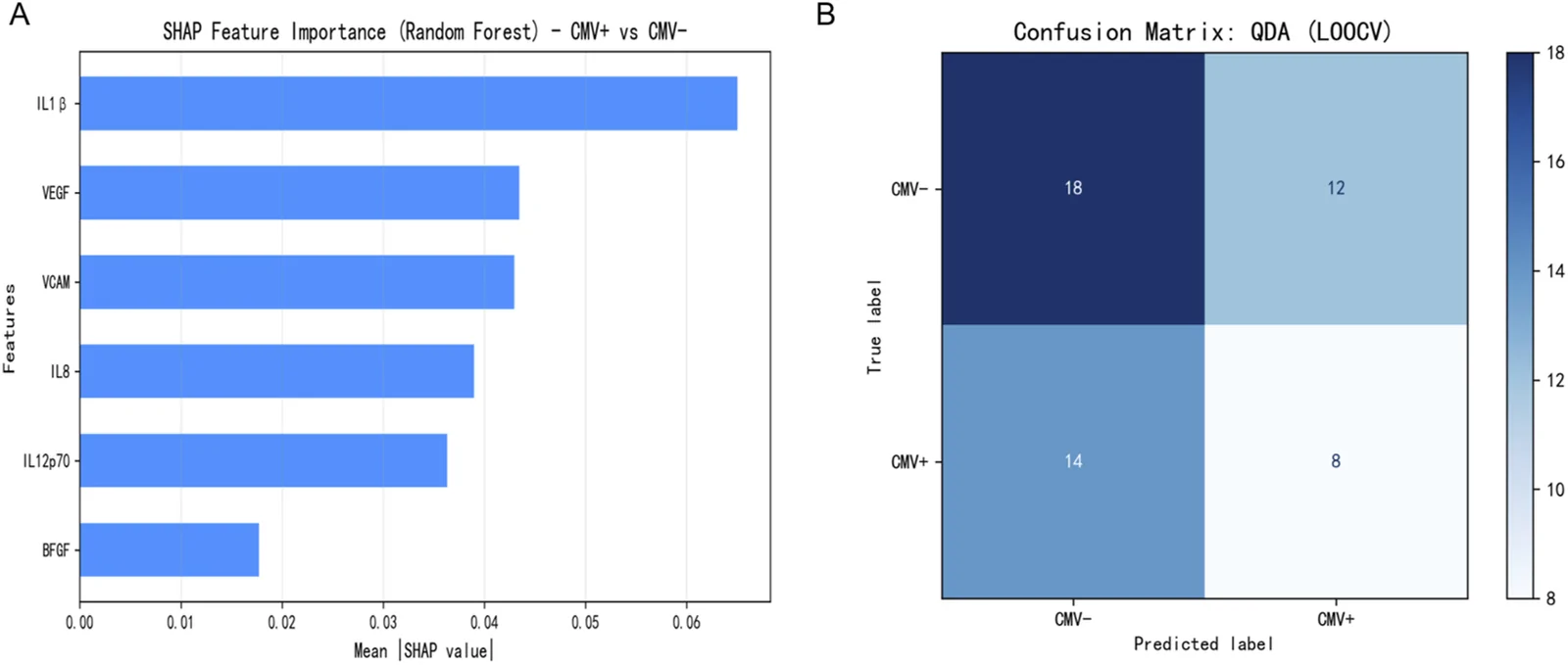
Feature importance and classification bias of the random forest model **(A)** SHAP value summary plot illustrating the feature importance of six input cytokines in the best-performing random forest model. Each point represents the SHAP value of a given cytokine for an individual sample. All cytokines had very low mean absolute SHAP values (highest value = 0.06 for IL-8), indicating negligible contribution to CMV status discrimination. The inconsistent direction of effects across samples further confirms the absence of systematic immune differences between the two subgroups **(B)** Confusion matrix of the QDA model for CMV status classification, evaluated by leave-one-out cross-validation. Rows represent true labels; columns represent predicted labels.

The confusion matrix of the best-performing QDA model (Figure 4B) shows that among 30 true CMV-negative samples, 18 were correctly classified and 12 were misclassified as CMV-positive (specificity = 60.0%); among 22 true CMV-positive samples, only 8 were correctly classified and 14 were misclassified as CMV-negative (sensitivity = 36.4%). The overall accuracy was 50.0%, nearly equivalent to random guessing. The model essentially defaulted to predicting the majority class for nearly all samples, a pattern consistent with the absence of a robust, detectable discriminative signal in the current dataset.

Learning curve analysis was conducted on the best-performing QDA model via 5-fold cross-validation, with progressively enlarged training set sizes (Figure 5). Training AUC remained at a consistently high level, starting near 1.0 with the smallest training set and decreasing only modestly to approximately 0.88 as sample size increased, indicating the model readily fit the training data. In contrast, cross-validation AUC rose slowly from around 0.40 to roughly 0.61 with expanding training set size, after which it reached a plateau and showed no further meaningful improvement. A substantial, persistent gap between training and cross-validation AUC was observed across all training set sizes, which is a classic signature of severe model overfitting. The learning curve suggests substantial overfitting and limited generalizability within the observed sample-size range. However, this analysis cannot exclude the possibility that larger cohorts or additional biomarkers may improve classification performance.

**FIGURE 5:**
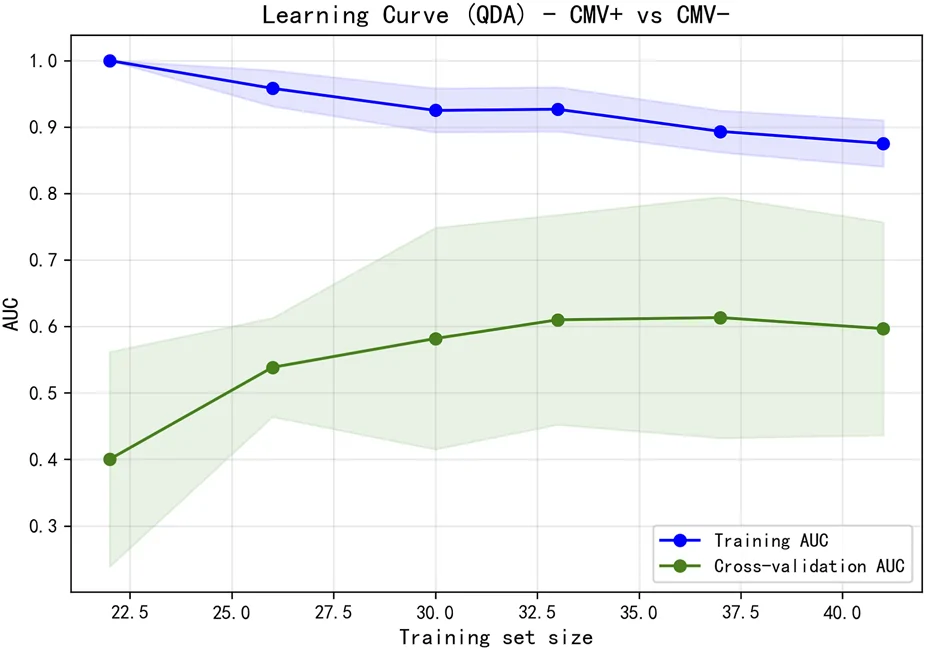
Learning curve of the quadratic discriminant analysis (QDA) model for discriminating CMV-positive from CMV-negative Posner-Schlossman syndrome. The blue line with circular markers denotes training AUC, and the green line denotes cross-validation AUC, plotted against increasing training set size. Shaded bands represent the standard deviation of AUC estimates across cross-validation iterations. The persistent wide gap between training and cross-validation performance indicates severe overfitting, and the plateau of cross-validation AUC at approximately 0.6 reflects limited discriminative signal within the cytokine panel.

To address potential confounding from other pathogens in the CMV-negative group, we performed a sensitivity analysis excluding the 9 patients with non-CMV pathogens. Reclassifying CMV-positive (n = 22) versus completely pathogen-negative PSS (n = 21) using the QDA model yielded an AUC of 0.540 (95% CI: 0.410–0.670), nearly identical to the primary analysis. This confirms that the poor discriminative performance is not an artifact of etiological heterogeneity in the CMV-negative group.

Calibration curves were constructed to evaluate the reliability of predicted probabilities from five representative models: logistic regression, random forest, linear SVM, QDA, and naive Bayes (Supplementary Figure 1). All models deviated markedly from the diagonal reference line of perfect calibration. Logistic regression, random forest, and naive Bayes exhibited pronounced stepwise patterns, with predicted probabilities heavily concentrated at the binary extremes of 0 and 1 rather than distributed across the full probability spectrum. Linear SVM and QDA also showed substantial miscalibration, with no model producing probability outputs that closely tracked the observed proportion of CMV-positive cases. These findings indicate that the probability estimates generated by all evaluated models are unreliable and lack clinical interpretability. In line with the poor discriminative performance, the inadequate calibration further confirms that there is no well-defined decision boundary between CMV-positive and CMV-negative PSS based on the measured cytokine panel.

### 3.5 Correlation between cytokines, clinical parameters, and feature collinearity

Spearman rank correlation analysis was performed to explore associations between aqueous cytokine levels and clinical characteristics in PSS patients. Intraocular pressure showed significant weak-to-moderate positive correlations with IL-8 (r = 0.42, P = 0.002), IL-1β (r = 0.38, P = 0.006), and VEGF (r = 0.35, P = 0.011). No statistically significant correlations were detected between any cytokine level and age, disease duration, best-corrected visual acuity, or corneal endothelial cell density (all P > 0.05).

To rule out multicollinearity as a potential cause of the poor machine learning performance, pairwise Spearman correlation coefficients were calculated among the six cytokines used as input features (Supplementary Figure 2). Overall, inter-cytokine correlations were uniformly weak, with coefficients ranging from r = −0.17 to r = 0.32. The strongest association was observed between IL-8 and bFGF (r = 0.32), which remains well below the conventional threshold for clinically relevant multicollinearity. The near absence of strong inter-feature correlations confirms that multicollinearity is not a confounding factor in our analysis, and therefore supporting that overlapping cytokine distributions, rather than multicollinearity, account for the poor classification performance; however, true biological equivalence cannot be confirmed with the current sample size.

## 4 Discussion

In this study, we integrated unbiased mNGS pathogen detection, multiplex cytokine profiling, and advanced machine learning analysis to investigate the relationship between CMV infection and intraocular immune phenotypes in PSS. Our key findings show that no statistically significant or stable difference in aqueous humor cytokine profiles was detected between CMV-positive and CMV-negative PSS patients, even with multiple machine learning approaches. These findings are consistent with the hypothesis that CMV may act as an inflammation amplifier, but our data do not definitively establish its biological role, and alternative pathogenic models cannot be excluded.

### 4.1 Implications for the CMV inflammation amplifier hypothesis

The role of CMV in PSS pathogenesis has been the subject of decades-long debate. The consistent detection of CMV DNA in a substantial proportion of PSS aqueous humor samples led to the widely held view that PSS is primarily a viral anterior uveitis caused by active CMV infection (Chee, 2015). However, several lines of evidence have challenged this primary pathogen hypothesis. CMV is a ubiquitous herpesvirus that establishes lifelong latent infection in multiple ocular tissues including vascular endothelial cells, trabecular meshwork cells, and resident macrophages, meaning viral DNA detection does not necessarily indicate active, disease-causing infection (Sinzger et al., 1996; Reeves and Sinclair, 2022). Clinically, CMV-positive and CMV-negative PSS patients present with highly similar phenotypes, and antiviral therapy reduces but does not eliminate recurrences, suggesting viral infection is not the sole etiological factor (Chee and Bacsal, 2011; Huang et al., 2023).

Our findings provide new molecular evidence relevant to this longstanding debate. If CMV were the sole primary driver inducing a distinct virus-specific inflammatory program, we would expect a clearly distinguishable cytokine profile in CMV-positive patients. Instead, the absence of a detectable difference in the measured cytokine panel is compatible with the inflammation amplifier model, in which CMV reactivation exacerbates a pre-existing core inflammatory process. However, we acknowledge the current cross-sectional data cannot definitively prove this pathogenic model. If CMV were the primary driver of PSS pathogenesis, we would expect CMV-positive patients to exhibit a distinct cytokine profile reflective of virus-induced inflammatory pathways, such as enhanced type I interferon responses or specific chemokine profiles associated with viral infection. Instead, we observed no statistically significant difference in cytokine levels between the two subgroups after correction for multiple comparisons. Most critically, even advanced machine learning algorithms specifically designed to detect subtle multivariate patterns were unable to reliably distinguish CMV status, with all models performing barely better than random guessing. Learning curve analysis demonstrates unstable generalization within the current sample size range, and whether expanded cohorts could identify a subtle but stable cytokine signature remains to be investigated.

The inflammation amplifier model provides a coherent explanation for all our findings. In this model, PSS arises from a foundational dysregulation of anterior segment innate immunity centered on the trabecular meshwork. This underlying immune abnormality creates a pro-inflammatory tissue microenvironment that can be triggered by various stimuli, including stress, oxidative damage, or viral reactivation. In individuals with latent CMV infection, the permissive inflammatory environment triggers viral reactivation from latency. Once reactivated, CMV infection exacerbates local inflammation through direct cytopathic effects on trabecular meshwork and endothelial cells, and by further stimulating cytokine and chemokine production, creating a vicious cycle that amplifies disease severity, prolongs episode duration, and increases recurrence risk. The observed cytokine similarities are compatible with the hypothesis that CMV may amplify a shared inflammatory process in some patients; however, the present cross-sectional data cannot establish its causal role or rule out CMV as a primary pathogenic trigger in a subset of cases. This model aligns with recent findings from other forms of anterior uveitis, where herpesvirus reactivation has been shown to exacerbate pre-existing sterile inflammatory conditions rather than causing distinct disease entities (Wu et al., 2024).

An important consideration is that the CMV-negative subgroup is etiologically heterogeneous, containing other viral and bacterial infections as well as pathogen-negative cases. In theory, this heterogeneity could dilute a true CMV-specific cytokine signature. However, the sensitivity analysis showed no improvement in classification performance after removing other pathogen-positive cases, strongly suggesting that different infectious triggers converge on a shared final inflammatory pathway in PSS. This also underscores that broad-spectrum pathogen detection (such as mNGS) remains indispensable, as cytokine profiles alone cannot distinguish specific causative pathogens.

Notably, our analysis is restricted to 9 soluble protein mediators measured at a single time point. We cannot exclude the possibility that CMV-positive and CMV-negative PSS may differ in other unmeasured biological dimensions, including alternative immune signaling pathways, viral-host molecular interactions, cellular immune subset compositions, transcriptomic profiles, or dynamic temporal patterns of the inflammatory response. Therefore, our findings do not rule out a primary pathogenic role of CMV in a subset of patients.

### 4.2 Innate immune dysregulation as the core pathogenic mechanism

The presence of a substantial proportion of CMV-negative PSS cases confirmed by unbiased mNGS indicates that viral infection is not a prerequisite for disease development, and that intrinsic dysregulation of ocular innate immunity constitutes the core pathogenic basis of PSS.

The trabecular meshwork, long viewed as a passive filtration structure for aqueous humor outflow, is now recognized as a critical immune sentinel tissue of the anterior segment. Trabecular meshwork cells express a full repertoire of pattern recognition receptors, including Toll-like receptors and nucleotide-binding oligomerization domain-like receptors, and secrete a broad spectrum of cytokines, chemokines, and extracellular matrix modifiers in response to inflammatory stimuli (Baudouin et al., 2013; Patel and Forrester, 2021). In PSS, aberrant activation of trabecular meshwork innate immunity may occur even in the absence of exogenous pathogens, triggered by endogenous danger signals such as oxidative stress, cellular senescence, or mechanical stress. Activated trabecular meshwork cells secrete pro-inflammatory mediators including IL-8, IL-1β, and VCAM-1, which recruit circulating leukocytes to the trabecular meshwork, induce trabeculitis, and impair aqueous outflow facility, leading to the characteristic IOP elevation observed in PSS. The resulting elevated IOP further damages trabecular meshwork cells, releasing additional damage-associated molecular patterns and perpetuating a self-sustaining inflammatory cycle.

Beyond intrinsic dysfunction of the trabecular meshwork, vascular endothelial dysregulation represents another important component of the sterile inflammatory process in PSS. The elevated levels of VEGF and VCAM-1 detected in the aqueous humor of our cohort are consistent with previous clinical studies documenting systemic and ocular endothelial activation in PSS and other forms of glaucoma (Gasser and Flammer, 2013; Kim et al., 2020). VEGF increases vascular permeability, allowing plasma proteins and immune cells to extravasate into the aqueous humor, while VCAM-1 mediates firm adhesion of leukocytes to the vascular endothelium, promoting their migration into the anterior segment. Endothelial dysfunction may thus both initiate and amplify intraocular inflammation in PSS, creating a feedforward loop that exacerbates trabecular meshwork damage and outflow obstruction.

A converging point for these diverse upstream triggers is the activation of the NLRP3 inflammasome pathway. As a master regulator of innate immunity, the NLRP3 inflammasome mediates the maturation and secretion of IL-1β in response to both pathogen-associated molecular patterns from infectious agents and endogenous damage signals from stressed cells (Schröder and Tschopp, 2010). In PSS, inflammasome activation can be initiated by viral components in CMV-positive cases, or by endogenous danger signals from stressed trabecular meshwork cells in sterile CMV-negative cases. This common final downstream pathway explains why patients with different etiological triggers present nearly identical inflammatory cytokine profiles. Supporting this hypothesis, a recent experimental study demonstrated that NLRP3 inflammasome components are significantly upregulated in the aqueous humor of PSS patients, and that pharmacological inhibition of inflammasome activation markedly reduces cytokine production in cultured human trabecular meshwork cells (Li et al., 2023).

### 4.3 Clinical implications for PSS management

The findings of this study have several important implications for the clinical management of PSS, particularly with regard to diagnostic workup and therapeutic decision-making.

From a diagnostic perspective, the high degree of similarity in both clinical phenotype and cytokine profile between CMV-positive and CMV-negative PSS underscores the irreplaceable role of direct pathogen testing in guiding treatment decisions. Clinicians cannot reliably infer CMV status based on clinical presentation, inflammatory severity, or cytokine levels, and empiric antiviral therapy is not justified. Currently, mNGS is increasingly used in clinical practice for its ability to detect a broad spectrum of pathogens simultaneously, and its diagnostic performance for ocular viral infections has been validated in multiple large cohort studies (Wang et al., 2021; Wu et al., 2024). Compared with targeted PCR, mNGS offers the advantage of unbiased detection, enabling identification of rare or unexpected pathogens that may contribute to disease exacerbation. For PSS patients with recurrent attacks, severe presentation, or poor response to conventional steroid therapy, early aqueous humor mNGS testing is recommended to identify potential viral pathogens and guide targeted antiviral treatment.

In terms of therapeutic strategy, our findings support a stratified management approach based on virological status. For patients with confirmed CMV infection, combination therapy with systemic antiviral agents and topical corticosteroids remains the standard of care. Antiviral treatment targets CMV replication and may reduce its potential amplifying effect on intraocular inflammation, while corticosteroids suppress the shared core inflammatory cascade observed across all PSS patients. Long-term observational studies have shown that this combination regimen reduces the frequency of PSS recurrences by approximately 60% in CMV-positive patients, although it does not achieve complete cure, which is consistent with the amplifier model (Chee and Bacsal, 2011; Huang et al., 2023). Treatment decisions should continue to be based on direct pathogen testing and established clinical evidence, as treatment response was not evaluated in the present study. The clinical value of antiviral therapy for pathogen-negative PSS remains to be determined in prospective interventional trials.

Most importantly, the identification of a shared core inflammatory pathway across all PSS patients opens new avenues for developing universal immunomodulatory therapies that benefit all patients regardless of viral status. Among the upregulated cytokines detected in this study, IL-1β represents a particularly promising therapeutic target given its central upstream role in the inflammatory cascade. Biologic agents targeting IL-1β, such as anakinra and canakinumab, have demonstrated favorable efficacy and safety profiles in the treatment of various non-infectious uveitis entities (Bruckner and Pleyer, 2019; Zhao et al., 2023). A recent preliminary clinical trial reported that intravitreal injection of anakinra achieved rapid control of intraocular inflammation in patients with refractory PSS, with a significant reduction in recurrence frequency over 12 months of follow-up (Liu et al., 2024). IL-8, which showed a significant positive correlation with IOP in our cohort, may represent another potential therapeutic target, as inhibition of IL-8 signaling has been shown to reduce trabecular meshwork inflammation and improve outflow facility in preclinical glaucoma models (Chen et al., 2023). Targeting these shared inflammatory mediators may provide a more effective and mechanism-based treatment approach for all PSS patients, complementing or potentially replacing broad-spectrum corticosteroid therapy and reducing the risk of steroid-related adverse effects.

### 4.4 Study limitations

Several limitations of this study should be acknowledged. First, this was a single-center study with a relatively modest sample size, particularly for the machine learning analysis. While LOOCV helps mitigate the impact of small sample size, the learning curve analysis is restricted to the current sample size range and cannot rule out improved classification performance in larger cohorts or with broader biomarker panels. Standardized quality control frameworks, as established for ophthalmic artificial intelligence applications, would further enhance the reproducibility and clinical translatability of cytokine-based classification models in future research (Yang et al., 2025). Larger multicenter cohorts would be valuable to confirm the generalizability of our findings. Second, we measured only a limited panel of 9 soluble protein immune mediators at a single cross-sectional time point. It remains possible that other unmeasured biological features including additional cytokines and chemokines, lipid mediators, cellular immune cell subsets, viral-host interaction signatures, transcriptomic profiles, or dynamic temporal changes in the inflammatory response which may differ between CMV-positive and CMV-negative PSS patients. Therefore, our null finding on cytokine profiles cannot definitively exclude a distinct biological role of CMV in a subset of cases. Future studies using comprehensive multi-omics approaches and longitudinal sampling are warranted to further delineate CMV’s role in PSS pathogenesis. Third, we did not perform longitudinal sampling to evaluate dynamic changes in cytokine levels over the course of disease episodes or in response to treatment. Longitudinal studies would provide further insights into the dynamic relationship between viral reactivation and immune responses in PSS. Additionally, the cataract control group is significantly older than the PSS group. Age and cataract status may influence baseline aqueous humor cytokine levels, which could partially confound the comparison between PSS patients and controls. However, this age difference does not affect the core conclusion of the study, as age distribution is nearly identical between the CMV-positive and CMV-negative PSS subgroups. Future studies with age-matched non-inflammatory controls are needed to more precisely define the PSS-specific cytokine signature. Finally, our study focused on protein-level cytokine profiles in aqueous humor; future studies investigating cellular immunity, including immune cell subsets and their functional states, may provide additional layers of understanding of PSS pathogenesis.

## 5 Conclusion

In conclusion, this integrated study combining mNGS pathogen detection, multiplex cytokine profiling, and advanced machine learning analysis shows that no significant difference in intraocular cytokine profiles was identified between CMV-positive and CMV-negative PSS patients within the current sample size and 9-plex immune mediator panel. The overlapping cytokine profiles are consistent with the inflammation amplifier hypothesis, but our data cannot definitively confirm CMV’s exact pathogenic role, nor exclude CMV as a primary disease trigger in a subset of patients. Definitive determination of CMV’s causal role requires further longitudinal and functional investigations. The study emphasizes that direct pathogen detection remains indispensable for guiding individualized antiviral therapy in clinical practice, and identifies shared core inflammatory pathways as promising universal targets for developing novel immunomodulatory treatments that benefit all PSS patients regardless of CMV status. Future larger multicenter studies using multi-omics approaches are warranted to further elucidate the complex pathogenesis of PSS and develop more effective therapeutic strategies.

## Data Availability

The raw data supporting the conclusions of this article will be made available by the authors, without undue reservation.

## Supplementary material

SUPPLEMENTARY FIGURE S1 Calibration curves for predicted probabilities of CMV-positive status across five representative machine learning models. The dashed diagonal line represents perfect calibration, where the mean predicted probability matches the actual fraction of positive cases. Five models are displayed: logistic regression (blue), random forest (orange), linear support vector machine (green), quadratic discriminant analysis (red), and naive Bayes (purple). All curves deviate substantially from the reference line, indicating poor reliability of model-generated probability estimates.

SUPPLEMENTARY FIGURE S2 Spearman correlation heatmap of the six cytokines used as machine learning features, analyzed in all PSS patients (CMV-positive and CMV-negative combined). Color scale indicates the strength and direction of correlation: red represents positive correlation, blue represents negative correlation, and numeric values of correlation coefficients are labeled in each cell. Pairwise coefficients range from −0.17 to 0.32, reflecting generally weak inter-cytokine correlations and minimal multicollinearity among input features.
